# Calcined Oyster Shell Powder as an Expansive Additive in Cement Mortar

**DOI:** 10.3390/ma12081322

**Published:** 2019-04-23

**Authors:** Joon Ho Seo, Sol Moi Park, Beom Joo Yang, Jeong Gook Jang

**Affiliations:** 1 Department of Civil and Environmental Engineering, Korea Advanced Institute of Science and Technology, 291 Daehak-ro, Youseong-gu, Daejeon 34141, Korea; junhoo11@kaist.ac.kr; 2 Applied Science Research Institute, Korea Advanced Institute of Science and Technology, 291 Daehak-ro, Youseong-gu, Daejeon 34141, Korea; solmoi.park@kaist.ac.kr; 3 School of Civil Engineering, Chungbuk National University, Chungdae-ro 1, Seowon-Gu, Cheongju, Chungbuk 28644, Korea; 4 Division of Architecture and Urban Design, Institute of Urban Science, Incheon National University, 119 Academy-ro, Yeonsu-gu, Incheon 22012, Korea

**Keywords:** shell waste, calcination, calcium oxide, cement mortar, strength, shrinkage

## Abstract

The present study prepared calcined oyster shell powder having chemical composition and crystal structure of calcium oxide and lime, respectively, and investigated the fresh and hardened properties of cement mortar incorporating calcined oyster shell powder as an additive. The test results indicated that the hydration of calcined oyster shell powder promoted the additional formation of Ca(OH)_2_ at the initial reaction stage, thereby increasing the heat of hydration. In particular, the volumetric increase of calcined oyster shell powder during hydration compensated the autogenous shrinkage of mortar at early ages, ultimately leading to a clear difference in the shrinkage values at final readings. However, an excessive incorporation of calcined oyster shell powder affected the rate of C–S–H formation in the acceleratory period of hydration, resulting in a decrease in the compressive strength development. Meanwhile, the degree of flow loss was inconsequential and rapid flow loss was not observed in the specimens with calcined oyster shell powder. Therefore, considering the fresh and hardened properties of cement mortar, the incorporation of calcined oyster shell powder of approximately 3% by weight of cement is recommended to enhance the properties of cement mortar in terms of compressive strength and autogenous shrinkage.

## 1. Introduction

There has been consistent effort to recycle wastes including industrial by-products in order to accomplish a sustainable society through resources recycling. Oyster shell is a by-product of oyster farming, constituting approximately 90% of an entire oyster in a mass ratio, and hence generates a significant amount of waste [1,2]. In coastal regions of oyster producing countries, a great quantity of shells including oyster shell has been being disposed rather than being recycled [3]. In South Korea and other countries, disposal of the oyster shell requires transportation to a designated area since oyster shell is classified as an industrial waste by the waste control act [1]. In this respect, illegal dumping and burying of oyster shell have been frequently observed raising critical social issues. Belated treatment of oyster shell waste results in various environmental concerns such as shortage of disposal area, significant disposal cost, odor, disfigurement, and rainwater-induced coastal pollution [1,4]. Against these backdrops, appropriate recycling of oyster shell waste is necessary to provide efficient and ecofriendly means of treating these wastes.

The size and shape of oyster shell depends on several factors affecting its process of growth, such as temperature, degree of supersaturation of calcium carbonate, and roughness of sea wave, nevertheless, more than 90% of an oyster shell is composed of calcium carbonate with magnesium carbonate and calcium sulfate as minor components [2]. In fact, oyster shell is considered to be a safe and recyclable resource since it does not contain any harmful substance. An oyster shell exhibits a rough surface and is very porous having considerable voids [1]. Ground oyster shell has a comparable apparent volume to sand with higher permeability and has a specific gravity of around 2.0 [1]. Many attempts have been given to utilize the physical and chemical characteristics of the oyster shell to fabricate adsorbents for hazardous substances, water purifiers, soil remediation agents, fertilizers, and construction materials [1,4,5,6,7,8,9,10,11,12,13,14,15,16].

Previous studies pertaining to the recycling of oyster shell waste have mainly focused on the replacement fine aggregate for concrete production. The majority of studies on mortar and concrete employed oyster shells after washing, grinding and drying in order to remove foreign substances, while some studies proceeded with those calcined at temperatures above 500 °C to volatilize organic matters [3,17,18,19]. A study on the utilization of oyster shell as a fine aggregate replacement conducted by Yoon et al. reported that ground oyster shell aggregate did not significantly affect the compressive strength up to 40% replacement ratio by weight of total aggregate [1]. However, ground oyster shell has a high specific surface area and low mechanical strength compared to that of sand, leading to an adverse effect on the mechanical strength of concrete or mortar with increasing aggregate replacement ratio (5–20% by weight of sand) [13,17]. Furthermore, oyster shell incorporation can reduce the workability of fresh concrete due to its porous nature and increase shrinkage due to low restraining effect and high rate of water loss [3,17,18]. Therefore, sufficient considerations are required for obtaining the optimum content of oyster shell aggregate: Yang et al. and Li et al. suggested that replacement ratio of oyster shell to sand should be limited to a maximum of 20% by weight of sand [17,20]. Lertwattanaruk et al. substituted cement with pulverized oyster shell having a particle size smaller than 75 μm at 0–20% by weight of cement, and demonstrated a reduction in drying shrinkage and thermal conductivity, while observing a decrease in the compressive strength [7].

Most studies on the use of oyster shell as a constituent material for concrete have investigated the effect of ground oyster shell as a fine aggregate on the concrete properties. These attempts are highly applicable without technical difficulties, yet having a low technical value from the viewpoint of enhancement of concrete performance and effective utilization of waste resources. Meanwhile, the oyster shell can be converted into lime by calcination. It is expected that hydration cement incorporating such oyster shell-derived lime as an additive may differ from sole hydration of cement. It was noted in a previous study that the lime leads to volumetric expansion during hydration [21], nominating itself as a potential expansive cement additive. Currently available expansive cement additives are mostly biased toward sulfate–calcium–aluminate components [22,23], which requires clinkering processes. In this regard, the oyster shell-derived lime can be an ecofriendly and effective cement expansion additive. Despite numerous studies involving use of calcium oxide derived from calcined oyster shell [8,12], utilization of calcined oyster shell as an additive to cementitious materials has not been reported. The present study, therefore, produces calcium oxide powder through the calcination of oyster shell and investigates the effect of its incorporation in cement on the fresh and hardened properties of cement mortar including workability, heat of hydration, compressive strength, autogenous shrinkage, and microstructure.

## 2. Experimental Procedure

### 2.1. Materials and Sample Preparation

**Figure 1:**
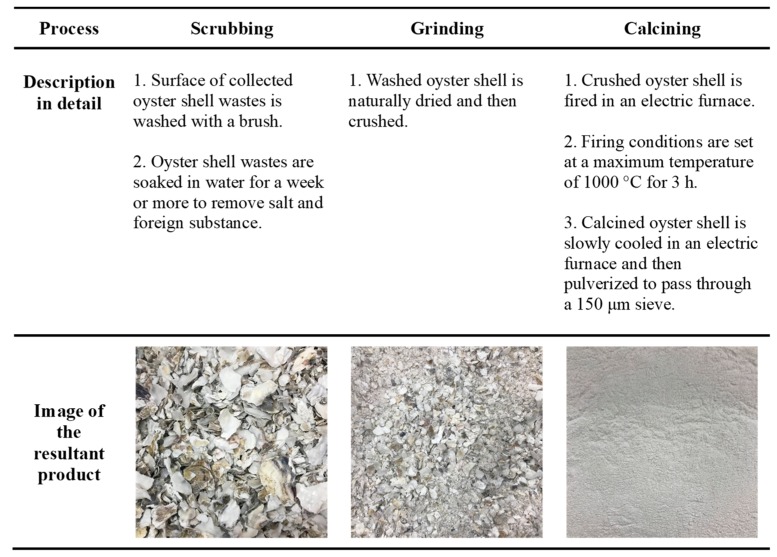
Manufacturing of calcium oxide powder through calcination of oyster shell waste.

Oyster shell wastes used in this study were sourced from the south coast of South Korea. The manufacturing process of calcium oxide powder through calcination of oyster shell waste is depicted in Figure 1. Surface of oyster shell was brushed and soaked in water for a week or more to remove salt and foreign substances. Washed oyster shell was naturally dried and then crushed. Crushed oyster shell was calcined in an electric furnace at 1000 °C for 3 h. Calcined oyster shell was slowly cooled down to room temperature in the electric furnace, and then pulverized to pass a 150-µm sieve.

**Figure 2:**
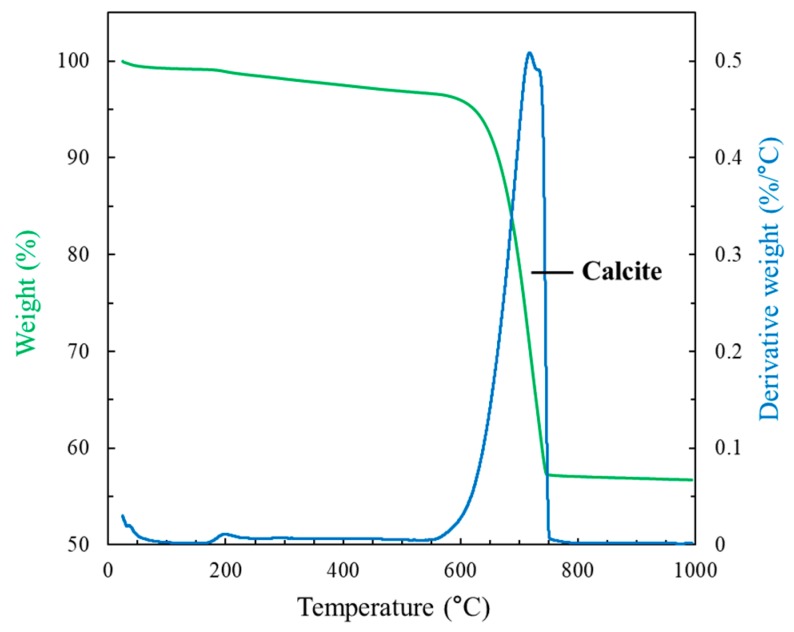
TGA/DSC result for a bulk oyster shell waste.

**Figure 3:**
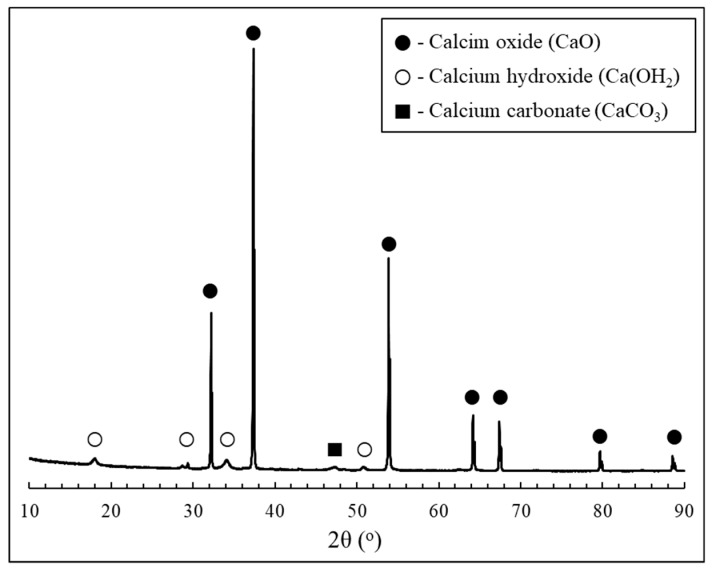
XRD pattern of calcined oyster shell powder.

Thermogravimetric analysis (TGA) and differential scanning calorimeter (DSC) results of a bulk oyster shell waste are shown in Figure 2. Since the oyster shell mainly composes of calcium carbonate, an endothermic peak at ~740 °C, corresponding to the decomposition of CaCO_3_ (Equation (1)), was observed in Figure 2. Thus, the calcination process of a bulk oyster shell is considered to be the conversion of calcium carbonate into calcium oxide. X-ray fluorescence spectrometry (XRF) and X-ray diffraction (XRD) results of obtained calcined oyster shell powder are presented in Table 1 and Figure 3, respectively. These confirmed that 98% of calcined oyster shell is comprised of pure calcium oxide having an identical crystal structure to lime.

CaCO_3_ → CaO + CO_2_ (1)

Cement was substituted with the calcined oyster shell powder at 0%, 3%, 6%, 9%, and 12% by weight of cement (Table 2). Mortar and paste specimens were prepared to avert the undesired influence of coarse aggregate. Type I Portland cement (density = 3.15 g/cm^3^ and Blaine fineness = 3.300 cm^2^/g) and standard sand (specific gravity = 2.34) were used to fabricate the specimens. Mortar specimens were used to evaluate compressive strength and shrinkage behavior, while paste specimens were used for microstructural and chemical analyses. The mix proportion of water:cement:sand of the mortar specimen was 1:2:3 and an identical water-to-cement (w/c) ratio was used for paste specimens.

Dry mixing of cement, calcined oyster shell powder and sand was performed for 3 min, followed by an additional 3 min of wet mixing with water. A portion of fresh mortar was used for table flow test. The remainder was poured into 50-mm-cubical and 100 mm × 100 mm × 400 mm prismatic molds. Specimens were sealed with plastic wrap for a day and demolded afterward. Specimens were cured in water till testing day. Entire casting and curing processes were carried out under a laboratory condition at temperature of 20 °C.

### 2.2. Test Methods

The experiments were systematically designed to explore the effect of calcined oyster shell additives on the fresh and hardened properties of cement mortar. The influence of calcined oyster shell powder on the hydration kinetics of cement was investigated by measuring heat of hydration using an isothermal conduction calorimetry (Tokyo Riko, Model MMC-511SV6, Tokyo, Japan). Paste samples with 0%, 6%, and 12% of oyster shell powder having w/c of 0.5 were used for isothermal conduction calorimetry test. The initial scanning rate, final recording rate, measuring period, and temperature of bath water for isothermal conduction calorimetry test were 30 s, 300 s, 72 h, and 20 °C, respectively. The effect of calcined oyster shell powder incorporation on the workability of mortar was explored by means of table flow test in accordance with ASTM C1437-15 [24]. Table flow was measure at 3, 10, 20, and 40 min after mixing to examine the flow loss over elapsed time. The table flow value was evaluated as a percentage of increased average base diameter relative to the original base diameter of fresh mortar [24]. The compressive strength was measured after 3, 7, 14, 28, and 56 days of curing using 50-mm-cubical mortar specimens. A compressive strength testing machine having a maximum loading capacity of 1000 kN was used to evaluate the average compressive strength of three replicates.

The microstructural characteristics of the cement mortar incorporating calcined oyster shell powder were evaluated by means of mercury intrusion porosimetry (MIP) and XRD. Autopore VI 9500 machine by Micromeritics Instrument Corporation (Norcross, GA, USA) was used for the MIP test. Test samples were collected from paste specimens at 56 days and were oven dried at 100 °C for a day before MIP test in order for the removal of free water. The pressure range used in the MIP test was 0.1 to 60,000 psi. The XRD analysis was conducted using a PANalytical 640c instrument by employing CuKα radiation at 40 kV and 30 mA, a scan speed of 0.2°/min and a scan range of 5 to 60°. Samples used for XRD analysis were pulverized paste specimens at 56 days which were oven dried beforehand at 100 °C for 24 h.

The incorporation of calcined oyster shell powder can affect both the cement hydration pathway and reaction products, which may lead to changes in dimensional stability. Autogenous shrinkage of mortar specimens was measured in this regard. An embedment type strain gauge (Micro Measurement, Model EGP-5-120) was installed at the center of a 100 mm × 100 mm × 400 mm mold before casting. After casting, mold was sealed with plastic wrap to prevent internal water from natural evaporation. Simultaneously, a portion of fresh mortar was used to determine final setting time by measuring the penetration depth by a vicat needle. Specimens for autogenous shrinkage were demolded when they reached a final setting as indicated by the vicat needle showing no penetration into the mortar. All faces of specimens were treated with aluminum tape and finished with plastic wrap to prevent undesired volumetric change attributed to drying shrinkage due to evaporation of free water. Specimens were then immediately placed in a thermo-hygrostat device. A plastic test bed with an oil layer was laid underneath the specimens to minimize the friction which might crucially affect the longitudinal movement of the specimens. The autogenous shrinkage behavior was recorded using a commercial data logger until changes in the shrinkage converged to a constant value.

## 3. Results and Discussion

### 3.1. Heat of Hydration

**Figure 4:**
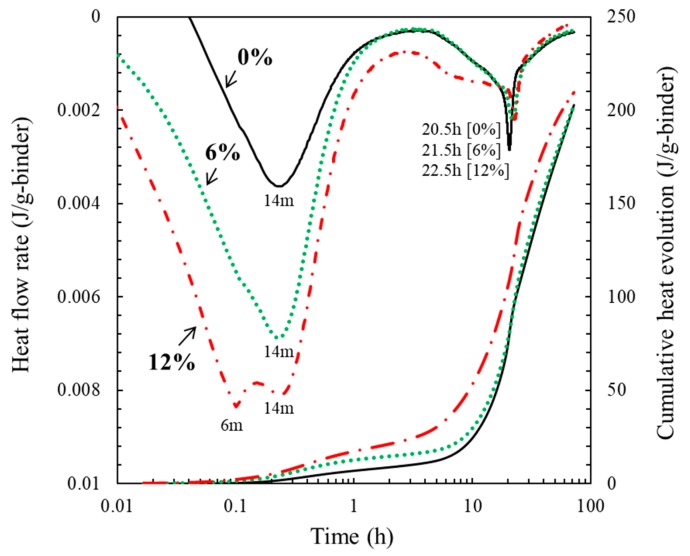
Isothermal conduction calorimetry of cement mortar incorporating calcined oyster shell powders of 0%, 6%, and 12%.

An isothermal conduction calorimetry of cement mortar incorporating calcined oyster shell powder of 0%, 6%, and 12% is shown in Figure 4. The cumulative heat evolution of the cement mortar incorporating calcined oyster shell powder of 0%, 6%, and 12% during 72 h of measurement was 202.7 J/g, 202.7 J/g, and 209.4 J/g, respectively. The heat evolution of specimen incorporating calcined oyster shell powder of 12% slightly increased as hydration proceeded, though incorporation of calcined oyster shell powder to cement did not significantly affect the cumulative heat evolution.

Meanwhile, heat flow rate curve displayed in Figure 4 showed a difference with different calcined oyster shell powder contents. Two distinctive peaks were identifiable on the heat flow rate curve, each observed at 14 min and 20.5 h, respectively, for control specimen, i.e., specimen incorporating oyster shell powder of 0%. The specimen incorporating oyster shell powder of 6% exhibited a first peak at 14 min, showing a relatively large amount of heat evolution compared to that of control specimen. A second peak appeared at 21.5 h, 1 h later than the control specimen. The amount of heat evolution at the appearance of second peak was slightly smaller than that of control specimen. The specimen incorporating oyster shell powder of 12%, on the other hand, presented two hump-like features at 6 min and 14 min, showing a clear difference with other specimens. The amount of heat evolution identified at 6 min and 14 min was higher than that of the specimens incorporating oyster shell powder of 0 and 6%, indicating that increasing calcined oyster shell powder content increased heat evolution at an early age. The specimen incorporating oyster shell of 12% showed a third peak at 22.5 h, 2 h later than control specimen. The amount of heat evolution at the appearance of third peak was somewhat smaller than that of other specimens, meaning that increasing calcined oyster shell powder content leads to a decrease in the heat evolution at a later stage and to a delay in the development of the very last peak. 

Heat of hydration of ordinary Portland cement can be categorized into five stages: (1) the initial reaction; (2) the induction period; (3) the acceleratory period; (4) the deceleratory period; and (5) the period of slow continued reaction [25]. It is well established that at the initial reaction stage when cement grains contact with water, dissolution of free lime yields Ca(OH)_2_ with an exothermic reaction [25]. A peak observed on the heat flow curve at 14 min, regardless of incorporated calcined oyster shell powder content, can be regarded as an exothermic reaction due to the hydration of free lime. The effect of increased calcined oyster shell powder incorporation on the early age heat flow was the presence of a peak appeared at relatively early age (6 min), as shown in the specimen incorporating oyster shell powder of 12% with an increase in the amount heat evolution. This phenomenon is possibly attributed to the fact that calcined oyster shell powder has a chemical composition identical to that of lime, acting like a free-lime in the cement. That is, CaO presented in the calcined oyster shell powder increased heat of hydration when dissolved in water by accelerating the reaction kinetic of Ca(OH)_2_. Meanwhile, the acceleratory period where a second peak appeared is generally affected by the rate of C–S–H formation [25]. In the present study, an increasing amount of incorporated calcined oyster shell had a slight effect on the appearing time and heat of hydration of the peak in the acceleratory period. The peak appearance time was slightly delayed and corresponding heat flow was decreased. It can be concluded from these observations that excessive incorporation of calcined oyster shell powder to cement has a potential to affect the rate of C–S–H formation due to the CaO composition in the calcined oyster shell powder.

### 3.2. Workability of Fresh Mortar

**Figure 5:**
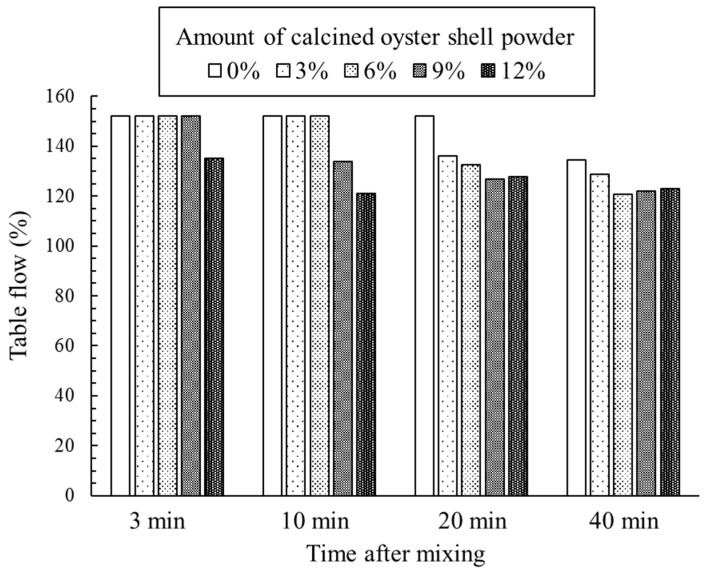
Table flow test results of cement mortar incorporating calcined oyster shell powder.

Table flow test results of cement mortar incorporating calcined oyster shell powder are shown in Figure 5. The table flow value of the control specimen maintained 152% until 20 min and showed a slightly reduced table flow value of 134% at 40 min. The table flow value of the specimens incorporating calcined oyster shell powder at 3 min was same as that of control specimen except for the specimen incorporating calcined oyster shell powder of 12%, which was lightly lowered to 135%. The addition of calcined oyster shell powder tended to accelerate the flow loss over time. As seen in the isothermal conduction calorimetry results, this observation was possibly attributed to the dissolution of CaO and consequent formation of Ca(OH)_2_ in the mixture of cement and calcined oyster shell powder which increased the consistency of fresh mortar during the contact with water. However, the average base diameter of the fresh mortars incorporating calcined oyster shell powder at 40 min was approximately 90% of that measured at 3 min, indicating that the calcined oyster shell powder addition did not lead to a rapid flow loss.

### 3.3. Compressive Strength

**Figure 6:**
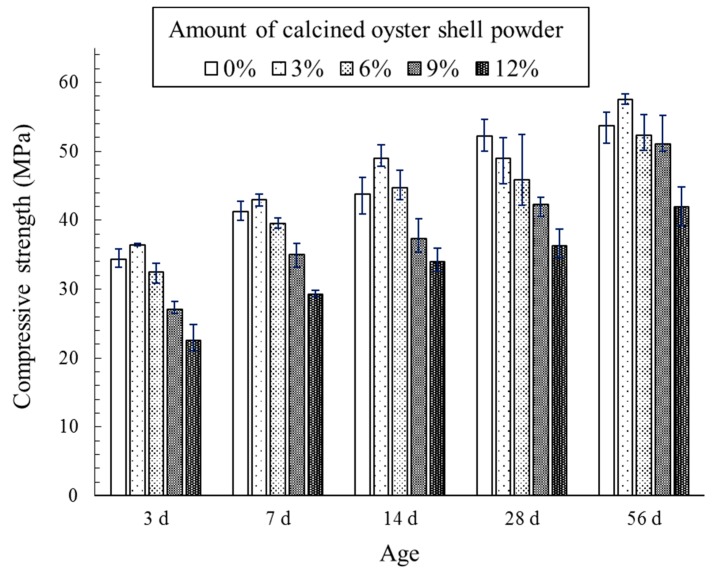
Effect of calcined oyster shell powder addition on the compressive strength development of cement mortar.

Effect of calcined oyster shell powder addition on the compressive strength development of cement mortar is shown in Figure 6. As provided in Figure 6, the amount of incorporated calcined oyster shell powder significantly affected the compressive strength development of cement mortar. The compressive strength of the specimen incorporating 3% calcined oyster shell powder at an early age (i.e., 3 days) was 36.4 MPa, exhibiting a modest strength increase compared to that of control specimen (34.4 MPa). The compressive strength of the specimen incorporating calcined oyster shell of 6% was similar to that of control specimen, while that of specimens incorporating calcined oyster shell powder of 9% and 12% were 27.1 MPa and 22.5 MPa, respectively, showing a notable decrease in the strength as compared to that of control specimen. This phenomenon is in a fair agreement with the test results presented in a previous study which demonstrated a linear strength loss with increasing shell content as a cement replacer regardless of the type of shell [7].

Compared with the control specimen, incorporation of calcined oyster shell powder of 3% positively contributed to the strength development with curing age. The specimen containing calcined oyster shell powder of 6% showed similar compressive strengths to that of control specimen throughout the measurements, yet the addition of calcined oyster shell powder more than 9% had a negative effect on the strength development. The compressive strength of the specimens incorporating calcined oyster shell powder of 0%, 3%, 6%, 9%, and 12% at 56 days were 53.7 MPa, 57.5 MPa, 52.3 MPa, 51.0 Mpa, and 42.0 MPa, respectively. These results suggest that the amount of incorporated calcined oyster shell powder should be limited to < 6% considering the workability and mechanical property of cement mortar.

### 3.4. Autogenous Shrinkage

**Figure 7:**
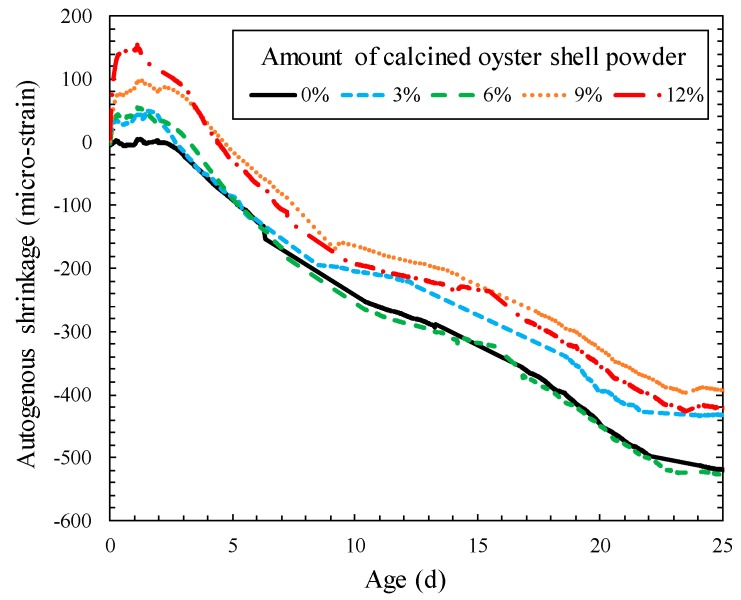
Effect of calcined oyster shell powder addition on autogenous shrinkage of cement mortar.

Effect of calcined oyster shell powder addition on the autogenous shrinkage of cement mortar is shown in Figure 7. The autogenous shrinkage value of the specimens incorporating calcined oyster shell powder of 0%, 3%, 6%, 9%, and 12% at 25 days were −520, −432, −527, −393, and −422 microstrain, respectively. Although the autogenous shrinkage value of the specimen incorporating calcined oyster shell powder of 6% was similar to that of control specimen, calcined oyster shell powder addition generally reduced the autogenous shrinkage of cement mortar. The autogenous shrinkage value of the specimen incorporating calcined oyster shell powder of 9% was 25% lower than that of the control specimen.

On the one hand, the effect of calcined oyster shell powder addition on the cement mortar was clearly identified through volumetric changing behavior at an early age. The initial volumetric behavior of the specimens during the first three days showed an expansion proportional to the amount of incorporated calcined oyster shell powder. The maximum initial expansion of the specimens incorporating calcined oyster shell powder of 3%, 6%, 9%, and 12% were +50, +55, +99, and +155 microstrain, respectively. The autogenous shrinkage of cement mortar can be regarded as a sort of chemical shrinkage which occurs as hydration reaction proceeds. As the calcined oyster shell powder is mainly composed of CaO, the volumetric increase of more than 2.5 times during the process of producing Ca(OH)_2_ by hydration reaction induced expansion [26], thereby compensating the shrinkage movement of cement mortar. Hydration of calcined oyster shell powder took place relatively faster than any possible reactions in cement (even faster than the free lime in the cement), which accompanied the initial expansion of the matrix. However, the hydration of calcined oyster shell actively occurred at an early age became serene as curing proceeded; indicating that the volumetric change of the matrix was governed by the hydration of cement clinkers at a later age.

### 3.5. Microstructural Characterizations

**Figure 8:**
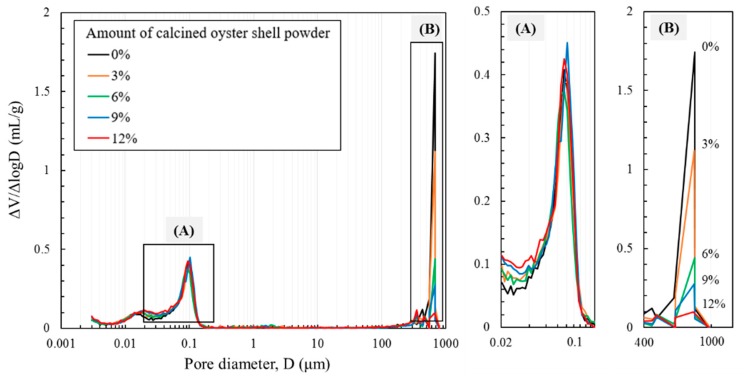
Pore size distribution (with pore size of 0.01 to 0.1 μm (**A**) and 100 to 1000 μm (**B**)) of cement paste incorporating calcined oyster shell powder measured by mercury intrusion porosimetry (MIP).

Pore size distribution of cement paste incorporating calcined oyster shell powder as measured by MIP is shown in Figure 8. A bimodal pore size distribution was observed in all specimens. Figure 8A displays pore size distributions with a pore size of 0.01 to 0.1 μm. The incorporation of calcined oyster shell powder did not lead to a difference in the development of micropores, meaning that the amount of micropore volume was unaltered with different calcined oyster shell powder contents. Figure 8B shows pore size distributions with a pore size of 100 to 1000 μm. The incorporation of calcined oyster shell powder obviously affected the pore characteristics in this pore region which corresponds to macropores. The amount of intruded mercury in the macropores region decreased as that of incorporated calcined oyster shell powder increased, showing a minimum level of symptom when 12% of calcined oyster shell powder was incorporated. Similar observation was reported in previous literatures showing that the ground seashells result in the segmentation of large pores, promoting the pore size refinement and packing of voids between cement particles [7,27].

**Figure 9:**
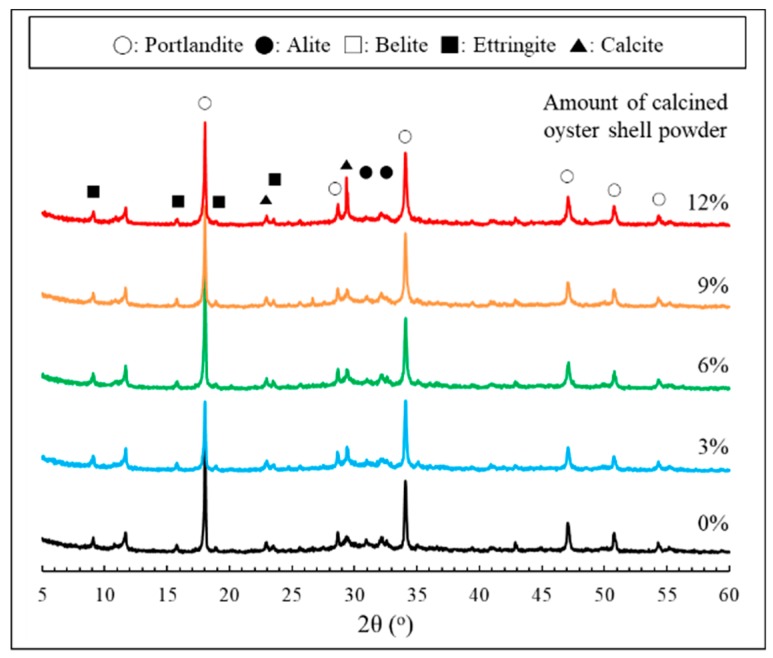
XRD pattern of cement paste incorporating calcined oyster shell powder.

XRD patterns of cement paste incorporating calcined oyster shell powder are shown in Figure 9. All samples displayed peaks associated with the presence of unhydrated clinker minerals, hydration products and carbonation products (i.e., alite, belite, portlandite, ettringite, and calcite). Meanwhile, peaks corresponding to the presence of CaO, as revealed in Figure 3, were unobservable in all paste samples, indicating that the incorporated calcined oyster shell powder was mostly consumed during hydration. The difference in the peak intensities featured on the XRD patterns with varying calcined oyster shell powder dosages was identified at around 29.4° 2θ. The appearance of this peak is generally attributed to the presence of calcite. Calcite (CaCO_3_) is a species of carbonation products precipitated by carbonic reaction of Ca(OH)_2_, in which its formation stems from the hydration of clinker minerals [28]. It should be noted that the XRD patterns of carbonated cement paste normally exhibit the development of peaks corresponding to calcite with simultaneous consumption of portlandite [29]. In this study, however, the XRD patterns of samples highly dosed with calcined oyster shell powder revealed the coexistence of portlandite with calcite. As explored earlier, this phenomenon is a result of the fact that a certain amount of portlandite, produced at an initial stage of hydration of calcined oyster shell powder, was consumed by natural carbonation, while portlandite was continuously produced by further hydration. The precipitation of calcite due to the carbonation of portlandite typically densifies the microstructure of cement paste [29], which is in close agreement with the MIP test results, which proves that increase in the amount of incorporated calcined oyster shell powder lead to a reduced quantity of macropores. However, it should be noted that the strength increase in the specimen incorporating calcined oyster shell powder of 3% was mainly due to the coupled effect of calcined oyster shell powder-induced pore structure refinement and precipitation of calcite, while the decrease in the strength of other specimens was highly associated with the reduction in the unit cement in their mixture proportion. Therefore, the reduced quantity of macropores in the specimens highly dosed with calcined oyster shell powder was much less likely to enhance the compressive strength than increasing the unit cement. This was also evidenced by the isothermal conduction calorimetry test results which pointed out the decreased rate of C–S–H formation in the specimens incorporating calcined oyster shell powder.

## 4. Conclusions

The present study prepared calcined oyster shell powder and investigated the effect of its incorporation on the properties of cement mortar in order to recycle the oyster shell waste which is generated in a large quantity annually. The oyster shell was calcined at 1000 °C using an electric furnace and 98% of the shell was composed of pure calcium oxide having a crystal structure of lime. Cement was substituted with calcined oyster shell powder at 0%, 3%, 6%, and 12% by weight of cement. Fresh and hardened properties of cement mortar including workability, heat of hydration, compressive strength, autogenous shrinkage, and microstructure were investigated. The main findings of the present study are listed below.

(1) In the hydration process of cement, calcined oyster shell powder incorporation increased the heat of hydration at the initial reaction stage due to the dissolution of CaO and formation of Ca(OH)_2_, demonstrating that the excessive incorporation of calcined oyster shell powder somewhat affect the rate of C–S–H formation in acceleratory period.
(2) The hydration of calcined oyster shell powder can induce the loss of workability by the increased consistency of fresh mortar due to the formation of Ca(OH)_2_, however the degree of flow loss was still inconsequential and rapid flow loss was not observed.
(3) The incorporation of calcined oyster shell powder had a significant influence on the compressive strength development of cement mortar. A small dose of calcined oyster shell powder (~3%) positively contributed to the compressive strength development. However, incorporation of the calcined oyster shell powder over 9% had a considerably negative effect on the compressive strength development.
(4) The incorporation of calcined oyster shell resulted in the expansion of matrix of cement mortar at an initial stage of hydration, thereby the compensating the length change by autogenous shrinkage.
(5) Considering the fresh and hardened properties of cement mortar, incorporating a small amount of calcined oyster shell powder at around 3% by weight of cement can contribute to the enhancement of the properties.

## Figures and Tables

**Table 1:** Chemical compositions of calcined oyster shell powder measured with X-ray fluorescence (XRF) spectroscopy as weight %.

wt.%	CaO	Na_2_O	MgO	Al_2_O_3_	SiO_2_	P_2_O_5_	SO_3_	Cl	K_2_O	MnO	Fe_2_O_3_	SrO
Calcined oyster shell powder	98.00	0.25	0.35	0.08	0.20	0.13	0.41	0.11	0.02	0.04	0.10	0.31

**Table 2:** Mixture proportion of mortar specimens used in this study expressed as mass ratio.

Substitution Ratio of Calcined Oyster Shell (wt.%)	Cement	Calcined Oyster Shell Powder	Sand	Water
0%	2	0	3	1
3%	2	0.06	3	1
6%	2	0.12	3	1
9%	2	0.18	3	1
12%	2	0.24	3	1
